# Fabrication of Nylon 6-Montmorillonite Clay Nanocomposites with Enhanced Structural and Mechanical Properties by Solution Compounding

**DOI:** 10.3390/polym14214471

**Published:** 2022-10-22

**Authors:** Ahmed M. Abdel-Gawad, Adham R. Ramadan, Araceli Flores, Amal M. K. Esawi

**Affiliations:** 1 Department of Mechanical Engineering, The American University in Cairo, AUC Avenue, P.O. Box 74, New Cairo 11835, Egypt; 2 Department of Chemistry, The American University in Cairo, AUC Avenue, P.O. Box 74, New Cairo 11835, Egypt; 3 Department of Polymer Physics, Elastomers and Applications Energy, Institute of Polymer Science and Technology (ICTP), CSIC, Juan de la Cierva 3, 28006 Madrid, Spain

**Keywords:** montmorillonite, nylon 6, nanocomposites, solution compounding, static melt annealing

## Abstract

Melt compounding has been favored by researchers for producing nylon 6/montmorillonite clay nanocomposites. It was reported that high compatibility between the clay and the nylon6 matrix is essential for producing exfoliated and well-dispersed clay particles within the nylon6 matrix. Though solution compounding represents an alternative preparation method, reported research for its use for the preparation of nylon 6/montmorillonite clay is limited. In the present work, solution compounding was used to prepare nylon6/montmorillonite clays and was found to produce exfoliated nylon 6/montmorillonite nanocomposites, for both organically modified clays with known compatibility with nylon 6 (Cloisite 30B) and clays with low/no compatibility with nylon 6 (Cloisite 15A and Na^+^-MMT), though to a lower extent. Additionally, solution compounding was found to produce the more stable α crystal structure for both blank nylon6 and nylon6/montmorillonite clays. The process was found to enhance the matrix crystallinity of blank nylon6 samples from 36 to 58%. The resulting composites were found to possess comparable mechanical properties to similar composites produced by melt blending.

## 1. Introduction

The production of polymer/layered silicate nanocomposites (PLSNs) with unique properties has been of interest to scientists and researchers. In 1989, researchers at the Toyota research center reported significant enhancements in the thermal and mechanical behaviour of nylon-6 after adding low contents of montmorillonite clay (MMT). This exposed an enormous research potential for this class of materials. Improvements in the mechanical, thermal, flame retardation, and gas separation properties as well as applications in biomedical and wastewater treatment fields have been frequently reported in the literature [1,2,3,4,5,6,7,8]. This strongly puts forward PLSNs as an attractive alternative to conventional micro-composites.

The mechanical and thermal properties of composites are generally dependent on the physico-chemical interaction between the matrix and the reinforcing phase. Due to the hydrophilic character of the silicate layers in the pristine clay (filler), and the organophilic character of most engineering polymers (matrix), interactions between the filler and the matrix are usually not favorable. This can be overcome by incorporating organic modifiers in the clay structure, which is usually achieved by ion exchange reactions where cations such as primary, secondary, tertiary, and quaternary alkylammonium or alkylphosphonium are used. Organic modification of the clay structure also leads to the increase in the distance between the silicate layers and inter-gallery spacing, which, in turn, facilitates the intercalation of the polymer matrix between the silicate layers [1,2,3,4,5,6,7,8,9].

It has been widely reported that significant improvements in properties can only be achieved for well-exfoliated and well-dispersed silicate layers [9]. Numerous studies addressed different preparation methods to improve the exfoliation and dispersion of the silicate layers [9,10,11,12,13,14,15,16,17,18,19]. The most common method for the preparation of PLSNs has been melt compounding. The key advantages are its speed and simplicity, as well as its compatibility with standard industrial techniques. This technique utilizes mechanical shearing forces, applied during extrusion or injection molding, to increase the inter-gallery spacing of the silicate layers, thus allowing the polymeric chains to diffuse into the clay galleries (intercalation). The technique can also lead to the separation of the silicate layers, resulting in the loss of their stacked form (exfoliation). However, complete exfoliation of the clay platelets was reported to be difficult to achieve [9]. The type of polymer affects the degree of intercalation or exfoliation, which are more easily achieved when organoclays are melt blended with polar polymers, such as polystyrene, polyamides, etc. but are a lot more challenging for apolar polyolefins such as polypropylene, polyethylene, etc. In addition, the change in melt properties, such as viscosity, upon the addition of the clay particles has been found to lead to polymer degradation under conditions of high shear rates, so careful selection of process parameters is crucial. Another challenge is that high melt temperatures can result in degradation of the organic modifier of the clay. Pre-exfoliation of clays prior to melt compounding by ultrasonication is common; however, as reported by Martinez-Colunga et al. [10], who prepared polyethylene–montmorillonite nanocomposites using different ultrasonic powers, restacking of the exfoliated nanolayers can occur after ultrasonication. To avoid the production of microcomposites, functionalization with polar monomers such as maleic anhydride (MA) or the addition of compatibilizers is usually utilized [16,20].

Some researchers have reported the possibility of the diffusion of the polymeric chains into clay galleries when samples are allowed to anneal above their melting temperature without being subjected to any shear forces, (sometimes referred to as unassisted exfoliation, melt intercalation or static melt annealing). For example, Vaia and Giannelis [21] indicated the possibility of the intercalation of organically-modified clays by polystyrene. In addition, Paci et al. [22] and Dennis et al. [23] reported full exfoliation for Cloisite30B/nylon6 using unassisted exfoliation. The degree of exfoliation was reported to depend on the annealing time needed for the polymer to diffuse into the clay galleries, which in turn depended on the molecular weight of the polymer and clay content. Investigations carried out with other clays presenting low compatibility between the polymer and the clay, such as Cloisite 25A, reported poor results. It was therefore suggested that polar interactions between the polymer and the clay are necessary for producing intercalated/exfoliated structures when shear forces are absent.

Solution compounding presents another approach for the preparation of PLSNs. The process entails the dispersion of the silicate layers in a solvent, which often results in swelling and then mixing the silicate solution with the dissolved polymer. Intercalation takes place when the polymer chains replace the solvent in the silicate layers galleries. Solvent removal is usually by precipitation in different non-solvents or by evaporation. Controlled removal of the solvent is crucial to the success of the process as any solvent residue can lead to polymer degradation during further processing. One of the benefits of solution compounding is that the agitation or stirring of the silicate solution prior to mixing with the dissolved polymer facilitates the separation and dispersion of the silicate layers in the final composite. Accordingly, the process can produce intercalated structures for polymers with little or no polarity. In addition, it has the advantage of not requiring high temperatures that may degrade the organic modifiers in the various clays. The process can be attractive for producing thin films with oriented intercalated clays. Selection of an appropriate solvent is a key requirement for the success of solution intercalation [9,24]. Many studies have focused on water-soluble polymers. For example, Strawhecker and Manias [25] prepared polyvinyl alcohol (PVA)/montmorillonite (MMT) nanocomposites, whereas Aranda and Ruiz-Hitzky [26] prepared polyethylene oxide (PEO)/Na^+^-MMT nanocomposites using a mixture of water and methanol as solvents. Similarly, Wu et al. [27] prepared intercalated PEO/Na^+^-MMT and PEO/Na^+^-hectorite nanocomposites. Some studies used non-aqueous solvents; for example, Ogata et al. [28] used chloroform to compound polylactic acid (PLA) with organically-modified MMT, but the process did not yield an intercalated structure.

Using solution compounding with engineering polymers has been reported in fewer studies. Yano et al. [29] prepared polyimide/MMT nanocomposites using a dimethylacetamide (DMAC) solution of polyamic acid and a DMAC dispersion of MMT modified with dodecylammonium cations. 12CH_3_-MMT, 12COOH-MMT, and Cloisite 10A-MMT were used in their study. X-ray diffraction (XRD) analysis showed that the type of clay strongly influenced the obtained structure with uniform dispersion, and the exfoliated structure was only observed for the nanocomposites with 12CH_3_-MMT. N-methyl-2-pyrrolidone was used as the solvent to prepare polyimide/MMT nanocomposites in another study [30]. A fully-exfoliated structure was reported when using low MMT contents, whereas nanocomposites with higher MMT contents were only partially exfoliated. High-density polyethylene (HDPE) and poly-dimethylsiloxane (PDMS) nanocomposites were also prepared by solution compounding [31,32]. Recently, Filippi et al. [20] reported that solution blending was unable to lead to intercalation in composites of ethylene copolymers and organically modified montmorillonite.

In addition to the study by Wu et al. [12], who prepared flame retardant polyamide 6/nanoclay/intumescent nanocomposite fibers through electrospinning using formic acid as a solvent, to the best of our knowledge, solution compounding was only employed in one other study of nylon6/layered silicate nanocomposites. In their work, Paci et al. [22] also prepared nylon6/Cloisite 30B nanocomposites using formic acid as a solvent. Their results showed that intercalation and even complete destruction of the silicate platelets stacking order is possible in cases of low filler content and small polymer/clay particles.

The current work focuses on the preparation of nylon-6/MMT nanocomposites by the solution compounding process. In addition to Cloisite 30B, which was investigated in the study by Paci as noted earlier, another MMT clay with a different organic modifier (Cloisite 15A) as well as unmodified clay (Na^+^-MMT) were also used to prepare the composites. The structural morphologies of the resulting composites, their mechanical properties as well as their crystallization behavior were explored.

## 2. Experimental Procedure

### 2.1. Materials Used

Nylon6 (3 mm pellets) was purchased from Sigma-Aldrich, USA. Natural sodium-based montmorillonite (MMT) clay (Cloisite Na^+^) and organically-modified sodium-based montmorillonite clays (Cloisite 15A and Cloisite 30B) were procured from Southern Clay Products, Inc., Austin, TX, USA. All of the clays used had an average particle size of 7 μm, as reported by the supplier. Table 1 presents the specifications of the clays used. Glacial acetic acid and 95% pure methanol were purchased from El Nasr Pharmaceutical Chemicals Co., Cairo, Egypt.

### 2.2. Sample Preparation

Composite samples (N6-Na^+^, N6-15A, and N6-30B) were prepared by solution compounding of Cloisite Na^+^, Cloisite 15A, and Cloisite 30B with nylon6. This entailed adding a suspension of each of the clays in 50 g glacial acetic acid to 50 g of nylon6 dissolved in 500 mL glacial acetic acid at 108 °C. This was followed by stirring until the mixture cools to room temperature. The amount of clay used was such that a final 5 wt% of clay in nylon6 was obtained after drying. Solvent evaporation was then carried out followed by flushing using methanol of any remaining acetic acid to give acetic acid-free composites. Those were dried at 90 °C until achieiving a constant weight. Blank samples of nylon6 without any clays (N6) were also prepared using the same routine to serve as reference samples.

The obtained powdered samples (composites as well as blanks) were then compression-molded at 240 °C for 5 min under a pressure of 65 MPa, to yield cylindrical samples 1 cm in diameter and 2 cm long. These were used for nano-indentation tests.

In order to investigate whether there are contributions to the attained morphologies from melt intercalation during the compression molding step, N6 samples were crushed into a fine powder and mixed with 5 wt% of the different MMT clays using Turbula^®^ T2F mixer, at 96 rpm for 1 h. The mixtures were compression-molded using the same conditions specified above to produce additional composite samples (referred to as “mechanically-mixed” or “static melt annealing” samples).

### 2.3. XRD

A D8 Bruker X-ray diffractometer, operated at 40 kV and 30 mA, and using Cu Kα (λ = 0.1542 nm) was used to record diffraction patterns at middle angle, MAXS, (diffraction angle 2θ = 2° − 10°), in order to monitor the basal reflection (d_001_). For each type of clay, four samples were analyzed: pristine clay (as-received); clay powder processed through the solution compounding routine without the addition of nylon6; nylon6 mixed with 5 wt% of Cloisite clay; and nylon6-Cloisite clay composite obtained by solution compounding. These four samples were analyzed in order to compare the effect of solution compounding on the basal reflection of the clays used. The Cloisite clay powder processed through the solution compounding routine without the addition of nylon6 served to divulge any effects of the solution compounding process on the clay basal reflection, and the blank N6 mixed with 5 wt% of Cloisite clay served to confirm that the significant decrease observed for the intensity of the d_001_ clay reflection was due to the exfoliation of the clay particles, and not merely to the lower clay content in the composite.

Two-dimensional wide-angle X-ray diffraction patterns, WAXS, (2θ = 8° − 35°) of pristine nylon 6 and the composites samples were obtained using a Micro Star rotating anode generator (Bruker, Karlsruhe, Germany) operating at 45 kV and 60 mA, and X-ray wavelength λ = 0.1542 nm. A Mar345 dtb image plate was used with a resolution of 3450 × 3450 pixels and 100 μm/pixel. The sample-to-detector distance was 250 mm. Diffraction patterns were analyzed using the FIT2D software [33]. All images showed isotropic diffraction rings indicating no preferred crystal orientation. The 2D diffraction patterns were azimuthally integrated to obtain intensity curves as a function of diffraction angle. The intensity profiles were fitted to several crystalline peaks and an amorphous halo as described in reference [34] and making use of the Peakfit program (Systat Software v.4.12, San Jose, CA, USA). The degree of crystallinity, *X_c_*, was calculated from the ratio of the area under the crystalline peaks to that of the total diffraction curve.

### 2.4. Nanoindentation

A Nanoindenter XP (Agilent, USA) was used for nanoindentation testing. Three arrays, each of twenty five indentations, were made at the top, middle, and bottom of the longitudinal cross-section of each sample. The distance between adjacent indentations was set to 100 µm in order to avoid the effect of interaction. A calibrated (Berkovich) tip was used under the continuous stiffness module (CSM). The indenter was loaded into the sample until a depth of 5000 nm. A constant strain rate of 0.05 s^−1^ was maintained throughout the test. Thermal drift corrections were not performed in order to avoid possible creep of the tested polymer. However, the test was not started until the thermal drift was stabilized below 0.05 nm/s. In addition, the test was carried out overnight after leaving the samples for three hours inside the nanoindenter to thermally equilibrate. Minimum and maximum calculation depths were set to 2000 nm and 4000 nm, respectively.

### 2.5. Melt Flow Index

The melt flow index (MFI) in g/10 min (235 °C, 2.16 kg) was measured with RAY-RAN Melt Flow Indexer (Ray-Ran Test Equipment Ltd., Nuneaton, UK) according to ASTM D1238-04c. MFI values were considered as an indirect measure of the viscosity of the nanocomposite. MFI testing was conducted on nanocomposite materials prior to compression molding. Consistency of the flow of the molten polymer was ensured during testing by excluding any extrudate containing voids. The die was cleaned between successive runs to prevent contamination. Three MFI values were recorded for each sample.

### 2.6. TEM

Samples were covered with a protective sputter coating of Au-Pd, then subjected to Focused Ion Beam (FIB) milling, using the lift-out method, to obtain electron-thin foil sections. TEM analysis was conducted on a Tecnai F20 (200 kV) TEM.

## 3. Results and Discussion

### 3.1. X-ray Diffraction

**Figure 1:**
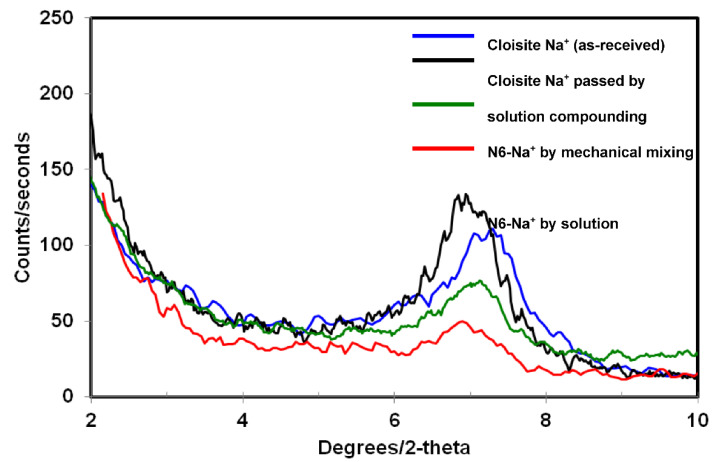
XRD diffraction patterns for the different Na^+^ and Na^+^ composite samples.

**Figure 2:**
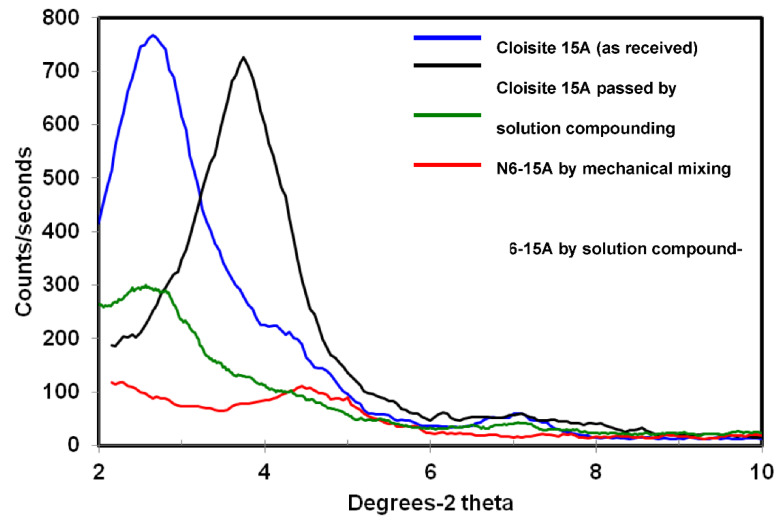
XRD diffraction patterns for the different 15A and 15A composite samples.

**Figure 3:**
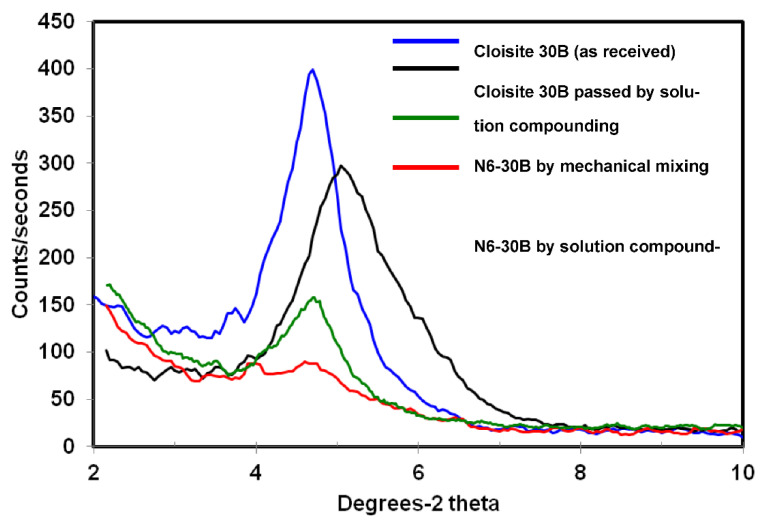
XRD diffraction patterns for the different 30B and 30B composite samples.

Middle angle X-ray diffraction spectra are shown for N6-Na^+^ (Figure 1), N6-15A (Figure 2), and N6-30B (Figure 3). Pristine Cloisite Na^+^ (as-received) exhibited a peak corresponding to a basal spacing of 11.7 Å. This peak shifted to a lower angle when Cloisite Na^+^ was subjected to the processing routine used for solution compounding without the addition of nylon6. The shift to a lower angle indicated a swelling of the clay structure as a result of stirring the clay in acetic acid. Mixed N6/Na^+^ powder showed a noticeable decrease in the intensity of the basal reflection peak as compared to the pristine as-received clay. This was due to lower clay content in the sample. This peak however appeared at the same two-theta angle as the pristine as-received clay. Upon compounding Na^+^ MMT with nylon-6, a significant decrease in the intensity of the basal reflection peak was observed. Furthermore, the peak appeared at a lower angle corresponding to a basal spacing of 12.8 Å. The decrease in the peak intensity of the compounded sample relative to the mixed sample (both containing 5 wt% clay) suggested partial exfoliation of the clay particles.

For Cloisite 15A (Figure 2), when subjecting the pristine clay to the solution compounding process without the addition of nylon6, the peak corresponding to the basal reflection shifted to a higher angle associated with the basal spacing of 23.6 Å. This decrease in basal spacing might be due to some destruction of the organic modifier. Lee and Char [35] argued that the organic modifier tails were more likely to bond to strongly acidic solvents, thus causing a collapse in the clay gallery spacing. Mixed N6/15A powder showed a noticeable decrease in the basal reflection peak intensity, due to the lower clay content in the sample, with the peak appearing at the same two-theta angle as the pristine as-received clay. Upon compounding with nylon6, the peak associated with basal reflection virtually disappeared, indicating significant clay exfoliation in the composite sample.

A similar trend was observed for Cloisite 30B samples, as shown in Figure 3 with the basal reflection peak shifting from a two-theta value, corresponding to 18.5 Å in the pristine as-received clay to a value corresponding to 17.5 Å for the clay subjected to the solution compounding routine, indicating a partial destruction of the organic modifier that could be due to rearrangement of its alkyl chains. Filippi et al. [36], who attributed a collapse in the interlayer spacing of Cloisite 30B to thermal degradation of the organic modifier after melt compounding 30B with polymer matrices, reported that the d-spacing collapse was reversed after dissolving the melt-compounded composite samples in appropriate solvents due to rearrangement of the alkyl chains of the clay modifier. Mixed nylon6-Cloisite 30B powders exhibited an XRD peak significantly lower in intensity due to the lower clay content and exhibiting a two-theta value similar to pristine as-received clay. Compounding Cloisite 30B with nylon6 led to the virtual disappearance of the basal reflection peak, indicating clay exfoliation in the composite.

**Figure 4:**
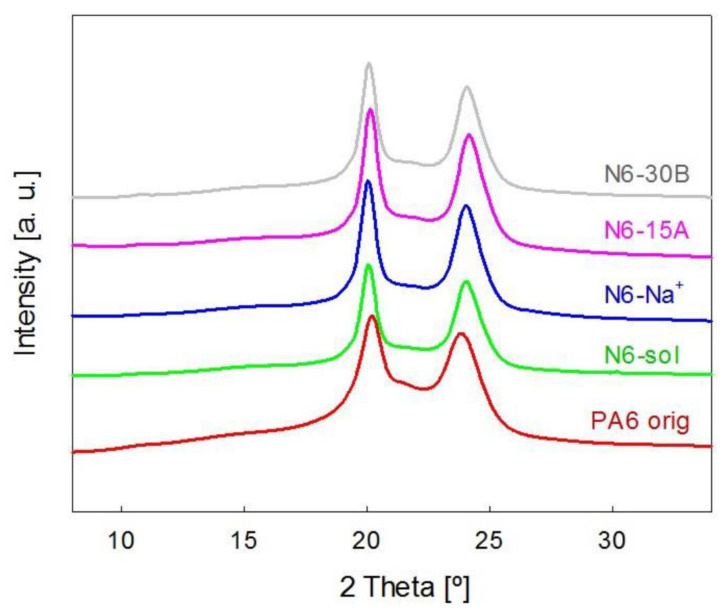
WAXS patterns of as-received and solution compounded nylon6, together with the solution compounded composites.

Figure 4 illustrates the WAXS patterns of as-received and solution compounded nylon6, together with the solution compounded composites. Results show that nylon6 crystals mostly adopt the monoclinic α-form, which is characterized by two strongly diffracting peaks at 2θ = 24° and 2θ = 20.5°. Only a very weak reflection at 2θ = 21.5° associated to the γ crystal structure can be discerned in the WAXS pattern of the nylon6 original sample. It is well known that nylon6 exhibits polymorphic structures, α and γ being the most common [37]. The α -form appears to be more stable than the γ structure partly due to enhanced hydrogen bonding interaction. Several authors have reported that melt compounding silicate layers into nylon6 induced the formation of the γ form [38]. Researchers have reported that slow crystallization or crystallization at high temperatures favors the α form whereas rapid crystallization or crystallization at low temperatures favors the γ form [39].

It is interesting to note that preparing samples using the solution compounding technique did not significantly alter the type of crystal structure obtained, with α crystal structure continuing to prevail. Furthermore, the addition of layered silicates did not cause a change in the crystal structure, in contrast to other nylon-layered silicates composites reported in the literature [37]. Another relevant aspect is that no preferential crystal orientation has been observed on any of the composites or neat nylon6. Indeed, the 2D WAXS images show isotropic diffraction rings for all samples and also the relative intensity of the two main reflections at 2θ = 24° and 2θ = 20.5° was found to remain constant. This behavior is different from that reported in the literature for nylon6/30B prepared by melt compounding in which the reflection at 2θ = 20.5° disappears upon the addition of clay, indicating a strong orientation of both the clay platelets and the polymer crystallites due to the shear stresses involved [22].

Concerning the amount of crystalline material, Table 2 shows similar *X_c_* values for all the solution compounded materials (approx. 0.58), including the neat polymer and regardless of the type of clay filler in the composite. However, PA6orig shows significantly lower values (0.36). It is also apparent that the crystalline peaks are wider in the case of PA6orig. This suggests that the crystal size is approximately the same for all the samples except for PA6 orig, which shows more limited crystal size.

The crystallization behavior of nylon6-clay nanocomposites processed by solution compounding has not been explored previously and therefore the current observations are reported for the first time. Results appear to indicate that the solution compounding process has a higher influence on the matrix crystallization behavior than does the polymer-silicate layers interaction.

### 3.2. Nanoindentation

**Figure 5:**
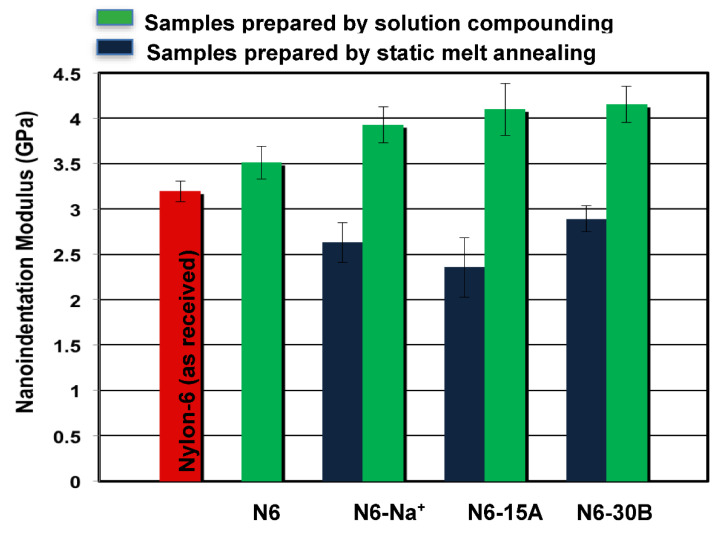
Average nanoindentation modulus values for the different composite samples.

**Figure 6:**
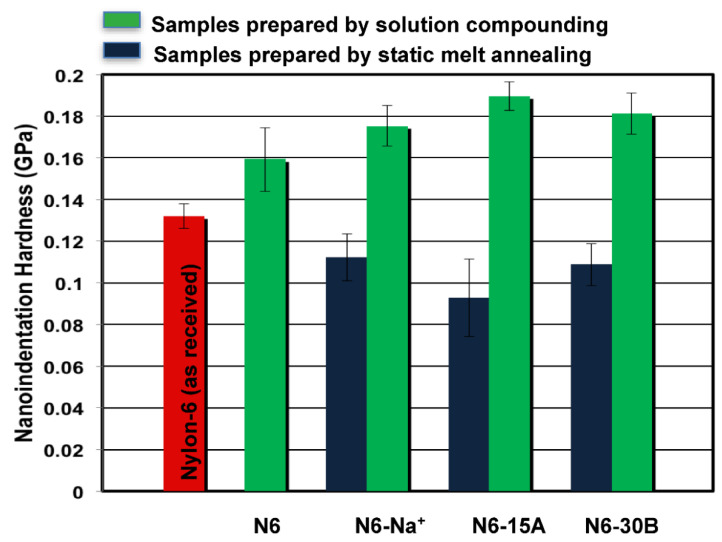
Average nanoindentation hardness values for the various samples.

Nanoindentation results, presented in Figure 5 and Figure 6, show an improvement in the mechanical behaviour (modulus and hardness) of the nylon-6 blank sample prepared by solution compounding, as compared to the as-received polymer. The increase in modulus of the blank polymer can be attributed to the higher amount of crystals with larger crystal sizes that develop during solution compounding, as indicated earlier based on the WAXS results. Dissolution of nylon-6 followed by solvent evaporation resulted in finer particles compared to the as-received pellets that melt at a faster rate. Since compression molding was carried out at 240 °C for 5 min, solution-compounded samples were annealed at this temperature for a longer period, which affected their crystallization behaviour and which is known to depend on melt annealing time, as observed in [40]. With regards to composite samples, all samples of nylon-6 compounded with the different clays showed improved modulus and hardness compared to the N6 blank samples as a consequence of the intrinsic higher modulus of the clays. On the other hand, static melt annealing produced composites with much lower mechanical properties compared to solution compounded ones, thus confirming that the observed enhancements in mechanical properties are due to the solution compounding process. Table 3 presents enhancements in nanoindentation modulus and hardness.

### 3.3. Melt Flow Index

**Figure 7:**
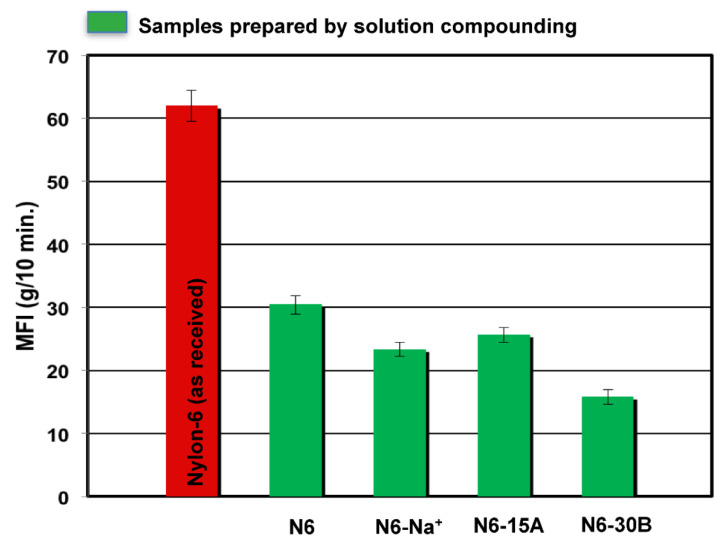
Melt flow index of nylon6/MMT nanocomposites.

The MFI results in Figure 7 show a significant decrease in MFI for the N6 blank sample compared to the as-received polymer. This might be due to the effect of melt annealing for longer time on the molecular weight of the polymer [40]. All composite samples exhibited lower MFI values with the N6-30B sample exhibiting the lowest value, i.e., highest melt viscosity which can be attributed to the better compatibility between the clay’s organic modifier and the polymer matrix. Similarly, the less pronounced decrease in MFI of the N6-Na^+^ and N6-15A samples is believed to be due to the lower compatibility between nylon6 and the clay layers (Na^+^ having no organic modifier and 15A having an organic modifier with lower compatibility with nylon6).

### 3.4. TEM

**Figure 8:**
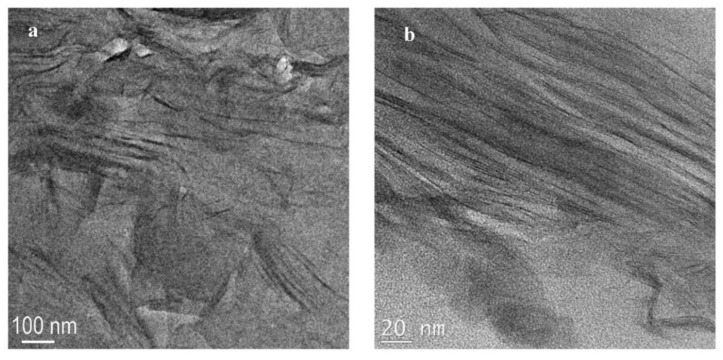
TEM micrographs for N6-Na^+^ prepared by solution compounding at low (**a**) and high (**b**) magnifications.

For the N6-Na^+^ composite, TEM images at low and high magnifications are presented in Figure 8a,b, respectively. The images reveal a composite with a mixture of intercalated and delaminated silicate layers. However, upon analyzing several areas of the sample, it became apparent that there are areas with a low density of silicate layers. The non-uniform dispersion is believed to be due to the absence of organic modifier in Cloisite Na^+^. This result agrees with the MAXS finding that showed a peak for the solution compounded composite that was quite similar in intensity and width to that of the mechanically-mixed composite.

**Figure 9:**
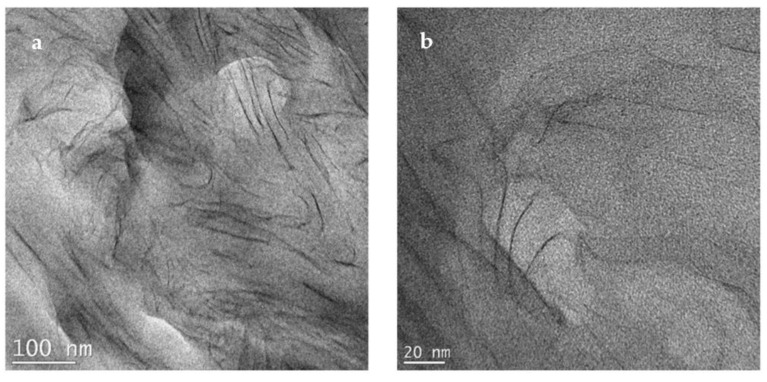
TEM micrographs for N6-15A prepared by solution compounding at low (**a**) and high (**b**) magnifications.

The TEM images of the N6-15A composite are presented in Figure 9 at low and high magnifications (Figure 9a,b, respectively) and show individual clay layers uniformly dispersed within the polymer matrix. However, similar to Na^+^, the examination of various areas across the sample revealed non-uniform clay distribution. A low intensity MAXS peak was detected, as presented in Figure 2, in agreement with the TEM observations.

**Figure 10:**
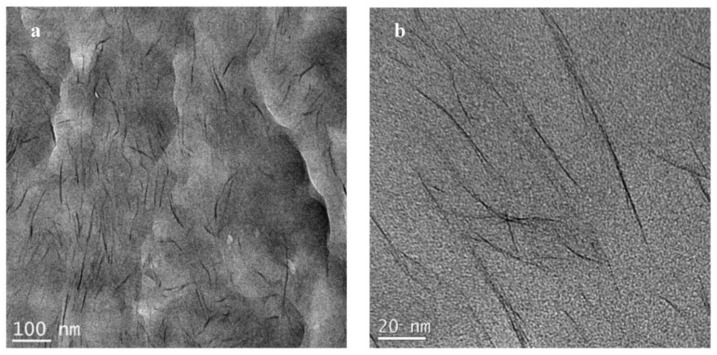
TEM micrographs for N6-30B prepared by solution compounding at low (**a**) and high (**b**) magnifications.

TEM images of the N6-30B samples are presented in Figure 10. Both low and high magnification images (Figure 10a,b, respectively) reveal a structure primarily composed of uniformly dispersed individual clay layers. This supports the MAXS results presented in Figure 3. The exfoliated layers coupled with the uniform dispersion—not observed in the other samples—elucidate the role of the type of organic modifier in influencing the morphology of the nanocomposites. We have reported in a previous study that the alkylammonium ions preserve the structure of the silicate layers in Cloisite 30B [41]. A similar observation was reported by Mani et al. for Cloisite 15A [42]. This challenges the common understanding that the increase in the gallery d-spacing due to the organic modifier makes the intercalation of the polymer chains easier. The organic modifier can therefore be considered to act in a twofold manner. On the one hand, it would bond with the polymer, facilitating its intercalation and the increase in the disorder of the clay structure, and on the other, it would stabilize the clay structure by maintaining the order of the layers. The polar organic modifier in Cloisite 30B is more likely to bond with nylon6 and thus increase the disorder in the N6-30B nanocomposite structure. This eventually leads to full exfoliation. For the case of the N6-15A composite with its non-polar organic modifier, the second factor is more dominant and thus exfoliation and a uniform dispersion of the clay layers is more difficult.

**Figure 11:**
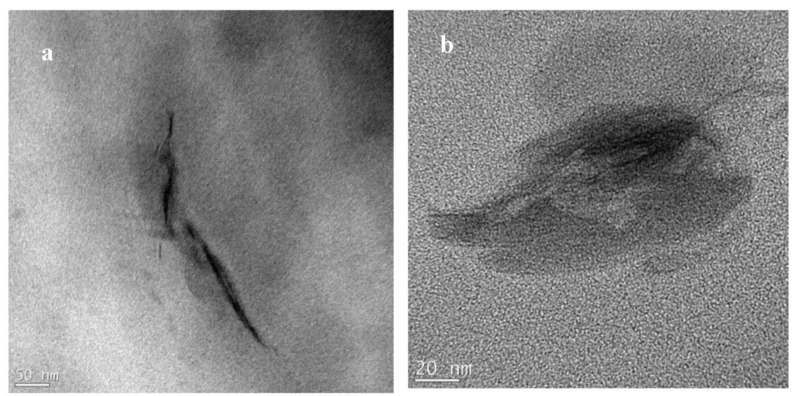
(**a**,**b**) TEM micrographs for N6-Na^+^ prepared by static melt annealing showing unevenly dispersed clay particles in different regions in the sample.

**Figure 12:**
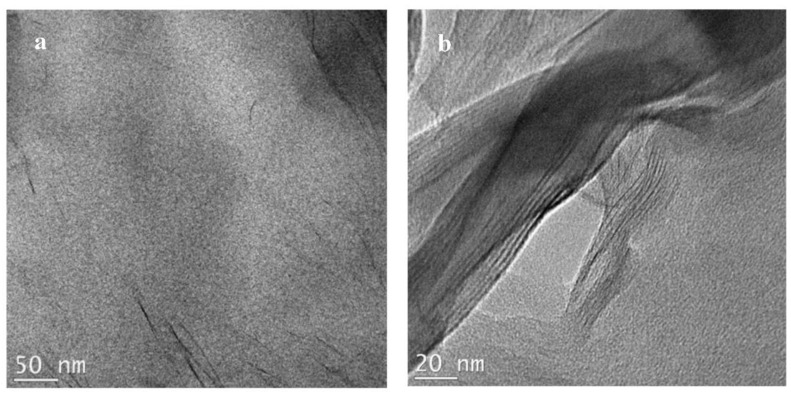
TEM micrographs for N6-15A prepared by static melt annealing showing some individual silicate layers (**a**) and intercalated particles (**b**).

**Figure 13:**
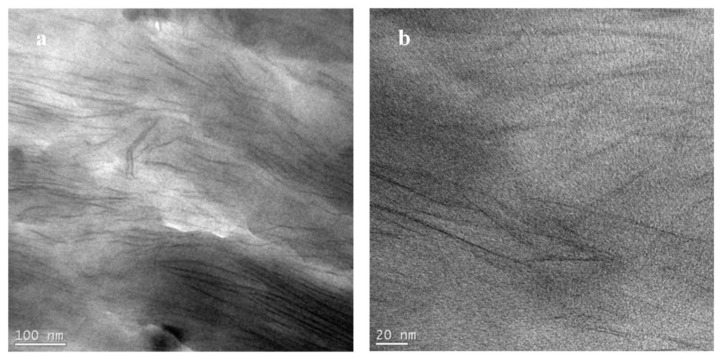
TEM micrographs for N6-30B prepared by static melt annealing showing evenly distributed silicate layers at low (**a**) and high (**b**) magnifications.

N6-clay samples prepared by the mechanical mixing of clay with N6 (i.e., static melt annealed samples), which were also analyzed by TEM. Results of N6-Na^+^ (Figure 11a,b) show unevenly dispersed clay particles and no isolated silicate layers. In addition, large areas of the examined sample did not have any clay particles, while in a few areas high densities of clay particles could be found. Composites of N6-15A showed some intercalation as well as some individual silicate layers (Figure 12a,b). However, similar to the N6-Na^+^ composites, large areas of the examined samples did not contain any clay particles, indicating poor dispersion. Composites of N6-30B (Figure 13a,b) showed better results with some areas having a high density of mostly evenly distributed silicate layers as well as other areas with very few layers. These observations are in line with the nanoindentation results that confirmed low mechanical properties for samples prepared by static melt annealing. This corroborates the fact that the intercalated/exfoliated structures of solution-compounded composite samples result from the solution mixing process.

With the exception of one study using solution compounding for the preparation of Cloisite 30B-nylon6 PLSNs [22], studies published on nylon6 PLSNs, in which testing of mechanical properties is reported, have predominantly used melt compounding [43,44,45,46,47,48,49,50,51,52,53,54]. Due to the limited use of solution compounding, it is important at this stage to compare the results obtained in the current investigation to those published in the literature pertaining to melt compounding. A comparison of our results to results from the literature is presented in Table 4. The search for those results has revealed that although many studies have been published on PLSNs, the general focus/theme has been the preparation method and how it influences the composite morphology. Testing of mechanical properties of nylon6-montmorillonite has been reported in only a few studies. Since enhancement of Young’s modulus has been the objective in these studies, we will focus on this property to give us a guide on how well our composites compare to others.

All results pertain to composites of nylon6 with 5 wt% clay. E_polymer_ is for nylon6 with the same process history as the composite.

Results in the table show that reported enhancements in Young’s modulus vary significantly from one study to the other, with one study reporting up to 88% enhancement upon the addition of only 5 wt% clay, whereas others report values as low as 2.5%. It is important to note that for organically modified clays, actual mineral content can vary depending on the amount of the organic modifier so although the results are reported for composites with 5 wt% clay, slight variations are expected. In addition, whenever different molecular weights of nylon6 are used (e.g., the study by Fornes [47]), a range is included for comparison. What is noticeable from the table is that the results of the current study compare favorably in terms of the absolute value of the Young’s modulus observed. This is believed to be due to the success of the solution compounding process in dispersing and exfoliating the different types of clays in the current study. It is also believed that the improved matrix crystallinity contributes to the observed enhancements, since it is known that the final properties of the nanocomposites will depend not only on the distribution of layered silicates but also on the crystal type, the degree of polymer crystallization, and crystallite morphology [37].

## 4. Conclusions

XRD and TEM investigations conducted in the current study confirmed that solution compounding facilitates the separation and dispersion of the silicate layers for cases of low/no polar interactions between the clays and the polymer. The results also revealed that solution compounding enhances the crystallinity for neat nylon6 from 36 to 58% and results in the more ordered and stable α type crystal structure. This enhanced the nanoindentation hardness and modulus. Additionally, solution compounding allowed a good dispersion of the 15A and 30B clays in the composites. The mechanical testing results compared favorably with published results in the literature for melt compounded samples and makes the process an alternative worth considering. It is also worth noting that solution compounded composites exhibited nanoindentation hardness and modulus values that are 60–110% higher than those produced by static melt annealing. Static melt annealing was found to be somewhat effective in the case of the N6-30B samples only, which further confirms that the observed exfoliated structures in the composites together with the associated enhancement of mechanical properties were brought about by the solution compounding process.

## 5. Patents

A US patent resulting from the work reported in the manuscript is listed below: Gawad, Ahmed Abdel M., Ramadan, Adham R., Esawi, Amal M. K., Solution blending process for the fabrication of NYLON6-montmorillonite nanocomposites, United States Patent 10100175, 2018.

## Figures and Tables

**Table 1:** Specifications of MMT clays.

Clay	Compatibilizer	Gallery d-Spacing d_001_ (Å)	Organic Content (% Mass)
**Cloisite Na^+^**	**-**	**11.7**	**-**
**Cloisite 15A**	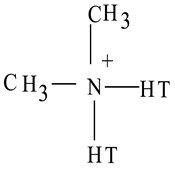 T = (~65% C18; ~30% C16; ~5% C14)	**31.5**	**43%**
**Cloisite 30B**	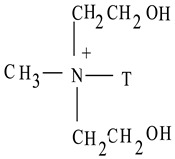 T = (~65% C18; ~30% C16; ~5% C14)	**18.5**	**28%**

**Table 2:** Degree of crystallinity by WAXS, *X_c_* of the various samples.

Sample	Degree of Crystallinity by WAXS, *X_c_*
PA6 orig	0.36
N6-sol	0.58
N6-Na^+^	0.57
N6-15A	0.58
N6-30B	0.58

**Table 3:** Nanoindentation modulus and hardness enhancements for the different composite samples.

	Modulus Enhancement		Hardness Enhancement	
	I ^◊^	II *	I ^◊^	II *
**N6-Na^+^**	12%	11%	10%	8%
**N6-15A**	17%	15%	19%	15%
**N6-30B**	18%	16%	14%	11%

^◊^ Percent enhancement values determined relative to the pristine polymer. * Percent enhancement values determined relative to the N6 blank.

**Table 4:** Comparing results of the current study to some reported Moduli of nylon6-montmorillonite clays.

Clay Type	E_polymer_ (GPa)	E_composite_ (GPa)	Preparation Method	Modulus Evaluation Technique	Reference
Cloisite 30 B	3.5	4.2	Solution compounding	nanoindentation	Current study
Cloisite 15A	3.5	4.15	Solution compounding	nanoindentation	Current study
Cloisite Na^+^	3.5	3.9	Solution compounding	nanoindentation	Current study
Cloisite 30B	3	3.7 (+23%)	Melt compounding	nanoindentation	[7]
Cloisite 15A	3	3.25 (+8.3%)	Melt compounding	nanoindentation	[7]
Cloisite Na^+^	3	3.2 (+6.7%)	Melt compounding	nanoindentation	[7]
organically modified clay (Nanomerw I.30TC)	1.06	2 (+88.7%)	Melt compounding	nanoindentation	[44]
Cloisite Na^+^	2.66	3.01 (+13.2%)	Melt compounding	Tensile testing	[45]
Organoclay SCPX 2004	2.66	3.66 (+37.6%)	Melt compounding	Tensile testing	[45]
Cloisite 30B	1.2	1.3 (8.3%)	Melt compounding	Tensile testing	[46]
MMT	1.11	1.93 (+73.9%)	(in situ polymerization)		[47]
MMT	2.82	4.2–4.8 (+59.6%)	Melt intercalation		[47]
Orgomodified MMT	1.99	3.12 (+57%)	Melt compounding	Tensile testing	[48]
Natural MMT	1.99	2.04 (+2.5%)	Melt compounding	Tensile testing	[48]
Organoclay	0.73–1.2	1.05–1.6 (+43%)	Melt compounding	Compression testing	[49]
OrganoMMT	2.9	4.1 (+41.4%)	Melt compounding	Tensile testing	[50]
